# Factors related to permanent disability employment on patients fewer than 62 years operated by open heart surgery

**DOI:** 10.1186/1749-8090-10-S1-A311

**Published:** 2015-12-16

**Authors:** R MartinezSanz, R Ávalos, L Perdomo, ME Alonso, F Benitez, JJ Jiménez, J Montoto, PC Prada, P Garrido, R de la Llana, M Brouard, JL Iribarren

**Affiliations:** 1 Complejo Hospitalario Universitario de Canarias, Tenerife, Spain; 2 Universidad de La Laguna, Tenerife, Spain

## Background

The quality of life after cardiac surgery greatly improves the reincorporation into the usual job, although not always in the same proportion.

## Objective

Our proposal is to identify factors that complicate the return to work after open heart surgery.

## Materials and method

All the patients younger of 62 years old who underwent open heart surgery between the years 2010 and 2012 were studied. Perioperative variables were collected: preoperative functional status, LVEF, type of intervention, type of job (employee or self-employed person) and permanent incapacity (PI) for employment through a review of their medical history.

## Results

A cohort of 204 patients was studied. Age 51 +/- 9 years; 156 (76,5%) were male; Logistic Euroscore (LE) of 5.1 +/- 8.4, LVEF 58 +/- 11. Surgery was 86 (42.2%) CABG, 79 (38.7%) valvular, 16 (7.8%) combined surgery and 23 (11.3%) others. 28 (13.7%) were self-employed. 15 of them already had a PI at the time of surgery. Patients with PI presented a LE of 6.7+/- 3.9 Vs. 11+/- 5 (p = 0.006); age 53+/-6 vs 48 +/-10 (p < 0.001), with no difference in LVEF. There were more PI among women (57%) than male 41% (p = 0.046). There was a higher percentage of valvular surgery in women. Higher number of CABG and valvular surgery was associated with PI (p = 0.015).

## Conclusions

Permanent work cessation activity after open-heart surgery was statistically determined with an older age, comorbidity, female gender and type of intervention. Valve surgery, the number of valves operated or higher number of bypasses increase the probability of PI.

